# Welcome to CTAD25 in San Diego!

**DOI:** 10.1016/j.tjpad.2026.100616

**Published:** 2026-06-10

**Authors:** Paul Aisen

**Affiliations:** Alzheimer’s Therapeutic Research Institute, University of Southern California, San Diego, California, USA

It is my great pleasure to welcome you to the 18th Clinical Trials on Alzheimer’s Disease Conference (CTAD25), taking place in San Diego, California from December 1–4, 2025. We are thrilled to host CTAD in San Diego this year, the 10 year anniversary of the founding of the USC Alzheimer’s Therapeutic Research Institute (ATRI).

Since its inception in 2008, CTAD has become a cornerstone event in Alzheimer’s disease (AD) clinical research — a meeting designed of AD therapeutic researchers to foster substantive, science-driven dialogue and international collaboration. By cultivating an intimate and collegial environment, CTAD continues to facilitate impactful partnerships and to advance the global AD research agenda.

The past two years have seen the full approval of the first disease-modifying treatments for AD, lecanemab and donanemab. Convincing evidence that they are disease-modifying include their mechanism of action, removing amyloid, the primary driver of AD pathology. Results of Phase 2 and 3 trials prove clinical efficacy, with a change to the slope of decline with separation from placebo that increases over time. Benefits persist after medication discontinuation, advantage over placebo is maintained after initiation of open-label treatment, and early treatment has a greater impact on progression than later treatment. We therefore expect that the benefits of these interventions will persist indefinitely with notable delays in markers of functional decline.

Lecanemab and donanemab slow progression by about 30%, a major advance in disease management. Several approaches to building on this progress are under way. More rapid and safer amyloid removal may be possible utilizing brain-shuttle methodology for antibody delivery to brain, as with trontinemab for Roche. Earlier treatment may be feasible. The AHEAD study, a public-private partnership funded by the National Institute on Aging, Eisai and philanthropy, is testing the efficacy and safety of amyloid removal with lecanemab in preclinical (pre-symptomatic, at least 40 centiloids on amyloid PET) and “pre-preclinical” (20-40 centiloids) AD. Lilly is testing donanemab in preclinical and prodromal AD. And Roche has shared plans to study trontinemab in preclinical AD. Dramatic progress in the validation of accurate plasma biomarkers of AD pathology have been greatly facilitating early intervention trial. Since co-pathologies increase with AD progression, combination approaches, for example targeting both amyloid and tau, may increase benefits at the symptomatic stages of disease.

With clarity in our understanding of AD pathobiology and the full spectrum of the disease, including the pivotal role of amyloid in initiating the process, we can now envision a path to primary prevention of AD. We may be able to refine plasma biomarker signatures that identify individuals with dysregulation of amyloid metabolism years before plaque accumulation in brain, allowing corrective therapy with secretase inhibiting or modulating drugs to eliminate that risk.

CTAD25 will also highlight results of studies of new therapeutic approaches for AD, including the EVOKE trials of the glucagon-like peptide-1 (GLP-1) agonist semaglutide.

This year ’s meeting carries special significance as we also celebrate the 10th anniversary of ATRI. The mission of ATRI is to advance AD therapeutics through innovation and collaboration, including public-private partnerships and commitment to widespread, comprehensive sharing of methods, complete trial datasets and specimens. At CTAD25, we celebrate our progress with the international community committed to defeating this disease.

CTAD25 will reflect the rapid advancements transforming Alzheimer’s diagnostics and therapeutics. The scientific program features five symposia, two roundtables, 74 oral communications, and more than 400 posters, all exploring the latest breakthroughs in AD research. Renowned clinicians and leaders from academia, research centers, patient coalitions, and industry will engage in dynamic discussions on topics ranging from disease-modifying agents and gene therapy to vaccines and multimodal interventions.

As we gather in San Diego, I invite you to join us in celebrating how far we’ve come and in charting the path forward together. Through continued scientific rigor, innovation, and collaboration, we can make the next decade one of transformational progress in Alzheimer’s disease research and prevention.

I look forward to welcoming you to CTAD25 in San Diego for what promises to be another inspiring and impactful meeting.

Warm regards.

## Declaration of Competing Interest

Dr. Aisen has research grants from NIH, the Alzheimer’s Association, Lilly and Eisai, and consults with Merck, Roche, Genentech, Abbvie, Biogen, Arrowhead, ImmunoBrain Checkpoint, AltPep, Bristol Myers Squibb, Johnson&Johnson and New Amsterdam.

